# Bioelectric Impedance Analysis Test Improves the Detection of Prostate Cancer in Biopsy Candidates: A Multifeature Decision Support System

**DOI:** 10.3389/fonc.2021.555277

**Published:** 2021-08-27

**Authors:** Riccardo Bartoletti, Alberto Greco, Tommaso Di Vico, Jacopo Durante, Vincenzo Ficarra, Enzo Pasquale Scilingo, Gaetano Valenza

**Affiliations:** ^1^Department of Translational Research and New Technologies in Medicine and Surgery, University of Pisa, Pisa, Italy; ^2^Department of Information Engineering & Research Center “E. Piaggio”, University of Pisa, Pisa, Italy; ^3^Department of Human and Paediatric Pathology “Gaetano Barresi” University of Messina, Messina, Italy

**Keywords:** prostate cancer diagnosis, bioelectric impedance analysis, prostate specific antigen (PSA), prostate-specific antigen density (PSAD), multiparametric MRI, computational statistical analysis

## Abstract

Prostate cancer (PCa) gold-standard diagnosis relies on prostate biopsy, which is currently overly recommended since other available noninvasive tools such as prostate-specific antigen (PSA) multiparametric MRI (mMRI) showed low diagnostic accuracy or high costs, respectively. The aim of the study was to determine the accuracy of a novel Bioelectric Impedance Analysis (BIA) test endorectal probe for the selection of patients candidate to prostate biopsy and in particular the clinical value of three different parameters such as resistance (R), reactance (Xc), and phase angle (PA) degree. One-hundred twenty-three consecutive candidates to prostate biopsy and 40 healthy volunteers were enrolled. PSA and PSA density (PSAD) determinations, Digital Rectal Examination (DRE), and the novel BIA test were analyzed in patients and controls. A 16-core prostate biopsy was performed after a mMRI test. The study endpoints were to determine accuracy of BIA test in comparison with PSA, PSAD levels, and mMRI and obtain prostate cancer (PCa) prediction by BIA test. The Mann-Whitney *U*-test, the Wilkoxon rank test, and the Holm-Bonferroni’s method were adopted for statistical analyses, and a computational approach was also applied to differentiate patients with PCa from those with benign disease. Combined PSA, PSAD, DRE, and trans-rectal ultrasound test failed to discern patients with PCa from those with benign disease (62.86% accuracy). mMRI PIRADS ≥3 showed a sensitivity of 83% and a specificity of 59%. The accuracy in discerning PCa increased up to 75% by BIA test (sensitivity 63.33% and specificity 83.75%). The novel finger probe BIA test is a cheap and reliable test that may help to improve clinical multifeature noninvasive diagnosis for PCa and reduce unnecessary biopsies.

## Introduction

**Figure 1:**
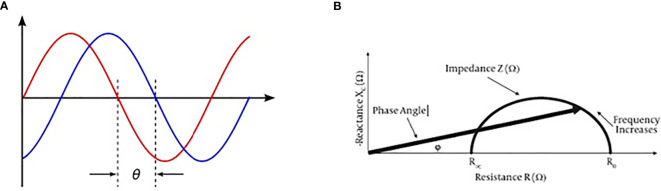
BIA test current voltage and phase angle definition. **(A)** Phase angle consists in a ratio between current out-of-phase and current phase, and it may be expressed by an angle value (ϕ). **(B)** Reactance and resistance measurement by BIA test may express a phase angle value.

Prostate cancer (PCa) diagnosis necessarily implies the use of prostate biopsy which is an invasive procedure burdened by potentially relevant complications such as bleeding and systemic infection. On the other hand, currently available noninvasive diagnostic tools seem to be unable to reduce the number of unnecessary biopsies. The decision-making process is mainly based on total prostate-specific antigen (PSA) values and the results of multiparametric MRI (mMRI) (1, 2). PSA levels alone are often unable to differentiate PCa from benign prostate hyperplasia (BPH) while the combination of total PSA and DRE, as well as the combination of PSA, DRE, and trans-rectal ultrasound (TRUS) improve the cancer detection rate to 50% (3, 4). mMRI in naïve patients remains of difficult application due to its high costs and the high number of men who need to be investigated in every day clinical practice, although its accuracy has been improved by the PIRADS V2 score classification (5). Therefore, there is a need for alternative noninvasive tools improving the selection of patient candidate for prostate biopsies. Previous studies on Bioelectric Impedance Analysis (BIA) revealed enthusiastic results mainly in patients with aggressive cancers (6, 7). Phase-sensitive instruments are able to simultaneously measure resistance (R), reactance (Xc), and provide the phase angle degree (PA). Very low PA values indicate cells with altered electrical activity due to different intracellular content, DNA, and water in cancers (8) (**Figure 1**).

Studies previously conducted on PCa characterization were limited by the applicability of proper probes on the prostate surface and the gland anatomic location inside the pelvic bone girdle (9–11). With the aim to reduce previous limitations in the applications of BIA test, we tested the performance of a novel endorectal probe in a series of consecutive patient candidate to prostate biopsy for suspicious PCa.

The objectives of the present study were to test the accuracy of BIA test to detect prostate cancer by analyzing three different parameters such as R, Xc, and PA, to evaluate if the proposed BIA methodology has to be further optimized to obtain clinically meaningful results and to develop a multifeature decision support system including BIA test parameters for the prediction of prostate cancer.

## Materials and Methods

### Patient Selection

All the patients who were candidates for a prostate biopsy for suspicious prostate cancer were consecutively and prospectively selected in the timeframe between March and September 2018. Presumptive diagnosis was based on persistently raised total PSA value (>4 ng/ml) and/or suspicion of cancer at DRE. Patients younger than 45 years and those affected by other neoplasms, electrolyte imbalance, and liver diseases were excluded from the study to avoid the risk of confounding factors. Moreover, patients with declared allergies to antibiotics and/or other compounds such as lidocaine, were excluded from the study. A total of 123 patients with persistently high total PSA levels and negative DRE underwent to mMRI and were classified in accordance with the PIRADS V2 system (5). Moreover, a group of young healthy volunteers were collected from a series of patient candidates to circumcision and selected with the same exclusion criteria. Healthy voluntaries were enrolled if total PSA value was **<**4 ng/ml, and no earlier history of prostate diseases or prostate-related symptoms was referred. Subjects included in the control group were not age-matched selected due to both the risk of developing familiar prostate cancer also in relatively young men (over45 years old) and the prostate growth that individually starts at the age of 30 but become usually symptomatic after 50 years of age (12). Patients who had undergone to prostate biopsy were age matched and had comparative risks of developing BPH or PCa. All patients selected for a prostate biopsy had a clinical suspicion of prostate cancer due to persistently increased PSA serum levels, and/or gland indurations at DRE, and/or familiar history of prostate cancer. Diagnosis of prostate cancer was performed on the basis of the pathological response after prostate biopsy. The study protocol was developed in accordance with the STROBE Statement and approved by Internal Review Committee (IRC) (1251/2017) and then registered (NCT03428087) (13). All patients and healthy volunteers provided their preliminary approval to participate in the study by signing an informed consent form.

For every patient, the following prebioptical parameters were collected: age, Body Mass Index (BMI), baseline total PSA (ng/ml), digital rectal examination (DRE), prostate volume estimated during TRUS examination, PSA density, and PIRADS score when available.

### The BIA Test and the New Endorectal Probe

**Figure 2:**
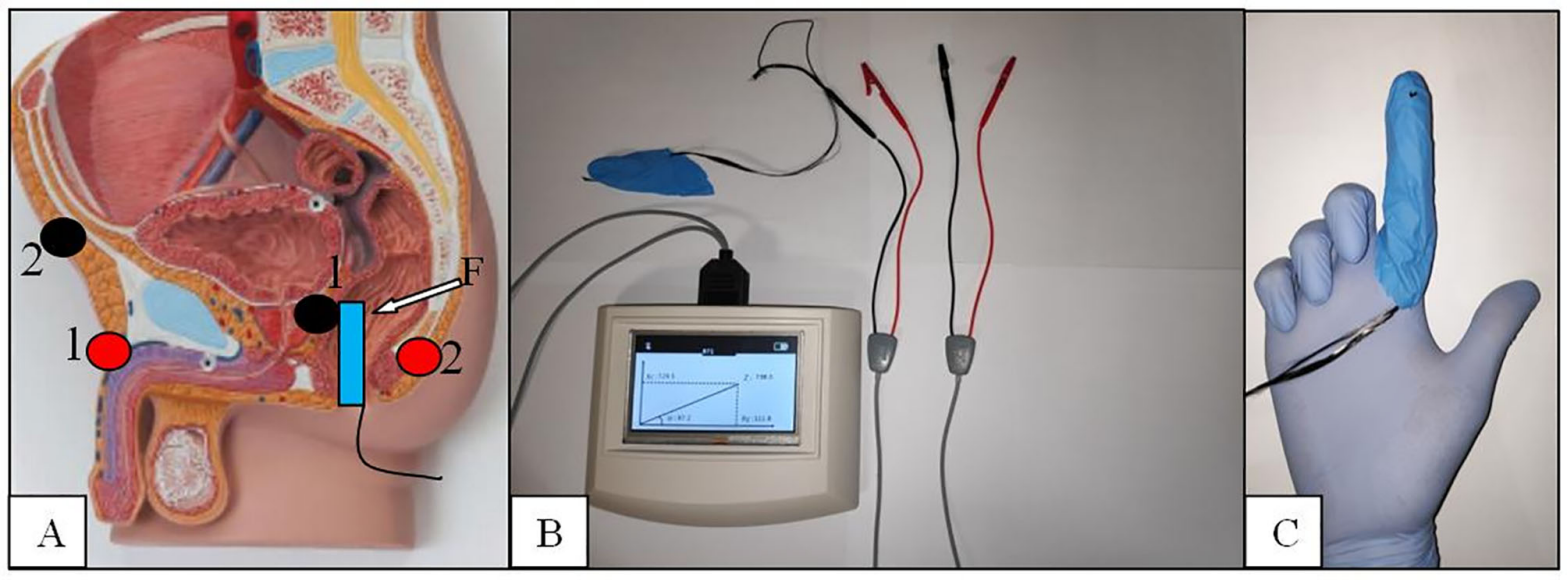
The BIA endorectal probe. **(A)** Electrodes placement red/black 1:1 and 2:2. **(B)** The BIA tester. **(C)** The finger probe with the carbon fibers placed at the tip of the second finger.

All patients underwent a BIA test using a new endorectal **“**finger probe**”** before to perform prostate biopsy. The Akern**’**s BIA tester is tested and validated instrument and was previously used to measure the biometric parameters (BIA 101 ASE**^®^**, Akern Srl, Italy) (14). The BIA test was provided with the patient placed in a left flank position as normally adopted for DRE and prostate biopsy procedures. The negative pole electrodes (red) were placed at the base of penile shaft and at the coccyx apex while the positive ones (black) were placed at half inch over the pubic bone and at the novel “finger probe”, respectively (**Figure 2**).

The electrodes placement was done to create a restricted electric field in the prostate area and get more reliable results in terms of sensitivity as demonstrated by other authors (9–11, 15). The novel probe was conceived to test the prostate tissue directly and consists of an electrode placement over a single rubber finger glove tip wearable over the rubber gloves normally used to detect prostate abnormalities. Carbon fibers are fixed at the tip of the probe, passed into the rubber finger, and connected to the “receptor”-positive pole BIA electrode. The use of flat and tender fibers other than rigid sensors was planned to allow an easy and sensitive concomitant palpation of the prostate gland. The BIA tester automatically calculated R, Xc, and PA, and different registrations have been made for the two prostate lobes. Because the location of possible cancer tissue is not known a priori, measurements from the BIA test were averaged between the two lobes for further analyses.

### Prostate Biopsy

Transrectal prostate biopsy was performed after combined antibiotic prophylaxis with ceftriaxone single shot and oral fosfomycin lasting for a couple of days and local anesthesia with 10% of 5 ml lidocaine. All the patients received a cleansing enema 2 hours prior the biopsy procedure and signed an informed consent to the procedure. In all cases, at least 16 cores were systematically taken with systematic criteria except for the patients with PIRADS V2 score **>**3 who received adjunctive cores in relation to the number of prostate gland sites described at mMRI (5). Prostatic cores were embedded in formalin solution and then analyzed by two uropathologists. The presence of cancer or other diseases were documented as well as the Gleason score classification in the case of cancer diagnosis confirmation.

### Statistical Analysis, ROC Curve, and SVM Classification

All BIA measures were normalized by dividing their value by the prostate volume, which was estimated during the TRUS examination. Therefore, the BIA test was not dependent by the volume of the prostate, which can be significantly affected by the presence of cancer.

Biomarker samples were statistically described using median and median absolute deviation (MAD) given the nonnormality of the majority of samples demonstrated using Kolmogorov-Smirnov tests (16). Accordingly, the Mann-Whitney *U*-test was used to statistically compare continuous variables from two different groups (e.g., patients vs. controls), whereas the Wilcoxon signed-rank test was used to compare differences in paired data (17, 18).

All *p*-values were corrected for multiple comparisons following the Holm-Bonferroni**’**s method (19).

A receiver operating characteristic (ROC) curve analysis was performed on PIRADS V2 scores gathered from 123 patients by pairing false-positive rates (1-specificity) and true-positive rates (sensitivity) at different PIRADS V2 score thresholds (5, 20).

To maximize a direct clinical applicability of the proposed study and move to a clinical evaluation at a single-patient level, we implemented a multifeature computational approach that takes into account all features, combine them through a particular mathematical function, and automatically estimate the multidimensional threshold to be used to make a clinical decision for the prediction of cancer presence. The computational methodology is quite common in the bioengineering field and is named support vector machine (SVM). We further extend the implementation of such a decision support system by integrating the so-called recursive feature elimination (RFE) approach. This scores each patient**’**s feature such that it is possible to rank and select the most informative clinical information for the automatic discrimination of patients with prostate cancer and BPH.

The proposed SVM model combining standard PCa biomarkers including BMI, PSAD, and PSA, together with BIA-related parameters was calibrated using data from *N* = 123 − 1 = 122 subjects and then tested using data from the *N*th subject for model validation. This validation procedure has been iterated *N* times following the so-called leave-one-subject out procedure (LOSO), where the calibration and validation sets randomly change at each iteration. The sensitivity and specificity of the proposed multiparametric approach are then calculated after N iterations, based on the observation of true positives/negatives and false positives/negatives.

## Results

**Figure 3:**
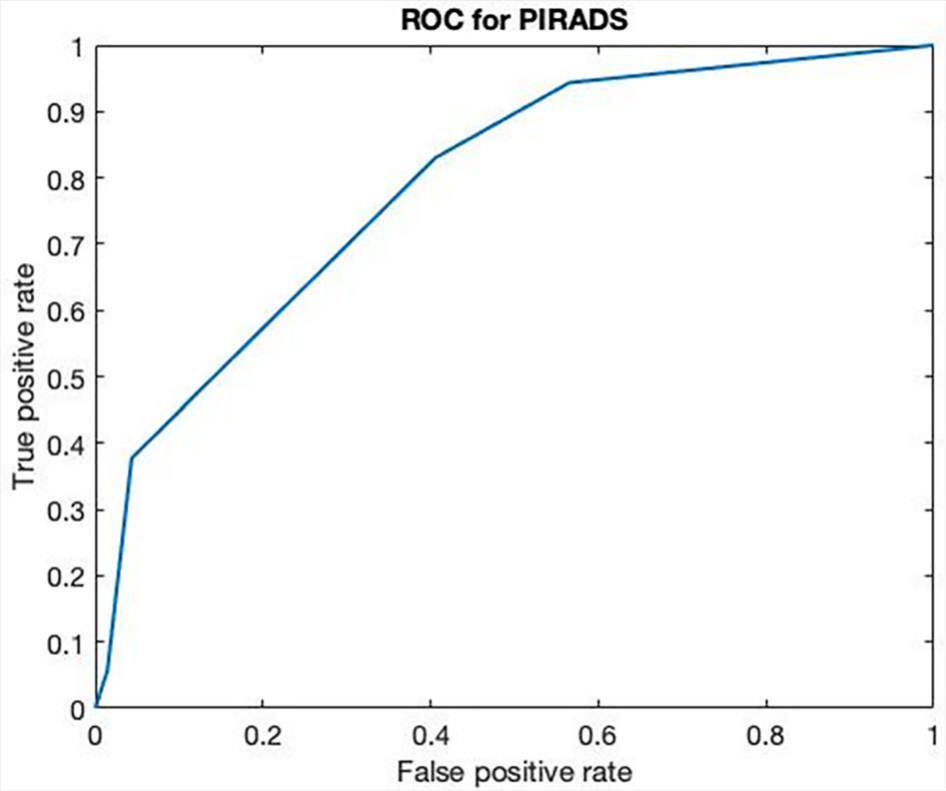
ROC curve of multiparametric MRI. ROC curve of multiparametric MRI on 123 patients with clinical suspicion of PCa (AUC 0.7893).

One hundred-forty men candidate to prostate biopsy for clinical suspicion of PCa were enrolled during the study period. No patients had relevant complication after the biopsy except for persistent bleeding in seminal fluid lasting for at least 1 month. Patients with total PSA levels of *<*4 ng/ml presented suspicion of cancer at DRE and 6 out 15 PIRADS *>*3 at mMRI. Cancer was detected in four out of 15 cases. PIRADS score *>*3 was found in three out of four subjects with PCa. Cancer was found in 31 out of 64 patients with total PSA between 4.1 and 10 ng/ml. PIRADS score *>*3 was found in 34 out of 58 cases, but only 22 of them presented association with PCa. Similarly, PCa was found in 21 out of 61 patients with total PSA *>*10 ng/ml. MMRI confirmed the presence of cancer in 18/25 patients although resulted positive in 31 out of 54 subjects. In 60 (42.8%) cases (median BMI, 26.25; IQR, 24.87–28.7), the biopsies resulted positive for prostate cancer while in the remaining 80 (57.2%) cases (median BMI, 25.75; IQR, 24.17–27.87), a nonneoplastic prostatic condition (BPH or inflammation) was diagnosed. ROC curve analysis performed on PIRADS V2 scores obtained from 123 mMRI of patients who underwent prostate core biopsy (no healthy volunteers have been included) showed a major threshold score or equal to 3* a* sensitivity of 83% and a specificity of 59% and VPP and VPN were 61% and 82%, respectively (**Figure 3**).

By analyzing patients with prostate cancer, in 21 (35%) cases, the disease involved a single lobe (11 the right side and 10 the left side). Conversely, in the other 39 (65%) cases, both lobes were involved by the tumor. Therefore, according to D*’*Amico risk classification, 31 (51.6%) patients were classified as low risk, nine (15%) as intermediate, and 20 (33.4%) as high risk (21). The 40 young healthy volunteers showed a median age of 37 years (MAD = 4) and a median BMI of 25 (MAD = 1.1). The median prostate volume was 19.23 cm^3^ (MAD = 6.5) with a median PSA density of 0.05 (MAD = 0.03). Comprehensive descriptive statistics of patients*’* characteristics stratified according to the biopsy results are reported in **Table 1**.

**Table 1:** Patients’ characteristics.

	PCa	BPH	Healthy controls
Patients (*n*.)	60	80	40
Age (years)	70 ± 5	69 ± 5	37 ± 4
BMI (kg/m^2^)	26.01 ± 1.51	26.15 ± 2.15	25 ± 1.10
Prebiopsy total PSA value (ng/ml)			
<4	4	11	35
4–10	31	33	0
>10	25	36	0
Prostate nodule/s at DRE (*n*. pts)			
Right	18	14	0
Left	10	7	0
Bilateral	8	4	0
Negative	24	55	35
Prostate volume at TRUS (ml)	41.48 ± 11.88	53.78 ± 13.30	19.23 ± 6.50
PSA density (PSA/volume)	0.25 ± 0.13	0.17 ± 0.07	0.05 ± 0.03
Prostate biopsy	YES	YES	NO
Low-risk PCa (*n*.)	31	–	–
Intermediate-risk PCa (*n*.)	9	–	–
High-risk PCa (*n*.)	20	–	–
Patients who underwent to prebiopsy mMRI (*n*.)	60	63	–
Patients with mMRI PIRADS ≥3 (*n*./%)	43/71.6	28/44.4	–
Patients with mMRI PIRADS ≥4 (*n*./%)	15/25	2/3.1	–

MAD, median absolute deviation.

Descriptive ranges are expressed as (median ± MAD).

Inferential statistics between patients with PCs, BPH, and controls are reported in **Table 2**.

**Table 2:** Statistical comparison between patients who underwent to prostate biopsy (cancer and BPH) and controls.

Feature	MEDIAN (PCa)	MAD (PCa)	MEDIAN (BPH)	MAD (BPH)	MEDIAN (HC)	MAD (HC)	*p*-Value	*p*-Value	*p*-Value
							PCa *vs.* HC	BPH *vs.* HC	BPH *vs.* PCa
AGE	70	5	69	5	37	4	**<0.001**	**<0.001**	n.s.
BMI	26.01	1.51	26.15	2.15	25	1.1	n.s.	n.s.	n.s.
PSA	8.985	2.535	9.11	3.915	0.87	0.45	**<0.001**	**<0.001**	n.s.
Prostate volume at TRUS	41.475	11.88	53.775	13.295	19.23	6.5	**<0.001**	**<0.001**	**<0.001**
PSA density	0.25	0.13	0.17	0.07	0.05	0.03	**<0.001**	**<0.001**	n.s.
R	41.425	10.325	47.125	12.45	34.25	10.95	n.s.	**<0.01**	n.s.
Xc	8.875	3.3	10.425	2.7	6.2	1.55	**<0.05**	**<0.001**	n.s.
PA	12.3	3.875	13.025	3.45	12.15	4.15	n.s.	n.s.	n.s.

n.s., nonsignificant p-value; MAD, median absolute deviation; BMI, Body Mass Index; PSA, prostate-specific antigen; TRUS, trans-rectal ultra sound; RES, resistance; REA, reactance; PHASE, phase angle; PCa, prostate cancer; BPH, benign prostate hyperplasia; HC, healthy controls.

Bold values indicate significant p-values lower than 0.05.

Comparing patients with PCa vs. controls, differences in age, PSA, prostate volume, and PSA density were found. Same statistical differences were found comparing BPH *vs.* controls. Concerning BPH *vs.* patients with PCa, significant differences were found in the prostate volume exclusively (p < 0.01); hence, PSAD analysis exclusively was retained for further analyses.

### Evaluation of BIA Test Parameters

**Table 2** summarizes and compares BIA test parameters gathered from patients with prostate cancer, benign prostatic disease, and healthy volunteers. While no significant differences between groups were found on the BIA parameter PA, significant differences were found in comparing BPH *vs.* controls using R (p < 0.01), as well as comparing controls *vs.* patients with PCa and controls *vs.* BPH using Xc (p < 0.05).

Concerning the statistical comparison between the three bioimpedance test measurements from the right and left sides of the prostate, we split the dataset in three subsets: (i) a subset of patients with right-sided prostate cancer (**Table 3**); (ii) a subset of patients with left-sided prostate cancer (**Table 4**); and (iii) a subset of patients with both-sided prostate cancer (**Table 5**).

**Table 3:** Median and MAD values of the bioimpedance features calculated on the right and left sides of the prostate in the right-sided prostate cancer group.

Feature	MEDIAN right side	MAD right side	MEDIAN left side	MAD left side	*p*-Value
R	46	9.6	46.9	9.45	n.s.
Xc	8.7	4.1	9.05	3.95	n.s.
PA	9.45	2.9	9.5	2.85	n.s.

The last column shows the results of the Wilcoxon signed-rank test for paired data between the left and right sides of the prostate (n.s., nonsignificant p-value).

**Table 4:** Median and MAD values of the bioimpedance features calculated on the right and left sides of the prostate in the left-sided prostate cancer group.

Feature	MEDIAN right side	MAD right side	MEDIAN left side	MAD left side	*p*-Value
R	40.5	7.9	40.8	9.55	**<0.01**
Xc	10.35	3.15	10	3.05	n.s.
PA	14.7	3.8	14.35	3.25	n.s.

The last column shows the results of the Wilcoxon signed-rank test between the left and right sides of the prostate (n.s., nonsignificant p-value).

Bold values indicate significant p-values lower than 0.05.

**Table 5:** Median and MAD values of the bioimpedance features calculated on the right and left sides of the prostate in the both-sided prostate cancer group.

Feature	MEDIAN right side	MAD right side	MEDIAN left side	MAD left side	*p*-Value
R	40.9	10.35	41.7	11.5	**<0.001**
Xc	8.4	2.7	8.5	2.75	n.s.
PA	12.4	3.9	11.55	3.5	**<0.05**

The last column shows the results of the Wilcoxon signed-rank test for paired data between the left and right sides of the prostate (n.s., nonsignificant p-value).

Bold values indicate significant p-values lower than 0.05.

It is worthwhile noting that the R of the right side of the prostate was significantly lower than the left side in the left- and both-sided cancer patient group. Moreover, PA of the left side of the prostate was significantly lower than the right side in the both-sided cancer patient group.

### Evaluation of a Multifeature Clinical Decision Support System for Diagnosis at a Single-Subject level

Using the parameter set comprising BMI, PSA density, PSA, AGE, R, PA, and Xc, we built an SVM multifeature computational model as described above and derived cancer recognition accuracy, sensitivity, specificity, positive, and negative predictive values (PPV and NPV) (**Table 6**).

**Table 6:** Feature ranking according to the RFE criterion.

Feature ranking
BMI
PSA density
R
PSA
PA
AGE
Xc

Feature ranges for healthy controls and PCa patients are reported in **Tables 1**, **2**.

For each feature, we performed a ROC curve analysis to evaluate the performance of each single feature in discriminating between patient with PCa from those with benign disease. From each ROC curve, we computed the area under the curve (AUC) and the related 95% confidence interval (95% CI AUC). According to the 95% CI AUC, we tested also if the AUC was statistically greater than 0.5, i.e., the chance performance value (AUC *p*-value).

Moreover, we identified the threshold cutoff point associated with the best accuracy (*Accuracy* Cut-off point) according to the Youden’s index (Sensitivity-Specificity-1) and the related sensitivity and specificity values (**Table 7**).

**Table 7:** Statistical analysis for each feature.

Feature	AUC	95% CI AUC	*Accuracy* cutoff point	Sensitivity	Specificity	AUC *p*-value
AGE	0.5597	0.4641-0.6553	75	0.825	0.267	0.110
BMI	0.51092	0.4141-0.6078	34.2	0.988	0.0333	0.413
PSA	0.49015	0.3931-0.5871	42.74	0.988	0.067	0.579
PSA density	0.62337	0.5311-0.7156	0.25	0.812	0.533	**0.00437**
R	0.52737	0.4308-0.6239	2.1185	0.925	0.167	0.289
Xc	0.50038	0.4034-0.5973	1.1439	0.975	0.0833	0.497
PA	0.55362	0.4578-0.6494	0.5291	0.925	0.200	0.136

The area under the curve (AUC) was calculated from the ROC curve as well as the related 95% confidence interval (95% CI AUC). According to the 95% CI AUC, the AUC was statistically greater than 0.5, i.e., the chance performance value (AUC p-value).

Bold values indicate significant p-values lower than 0.05.

**Figure 4:**
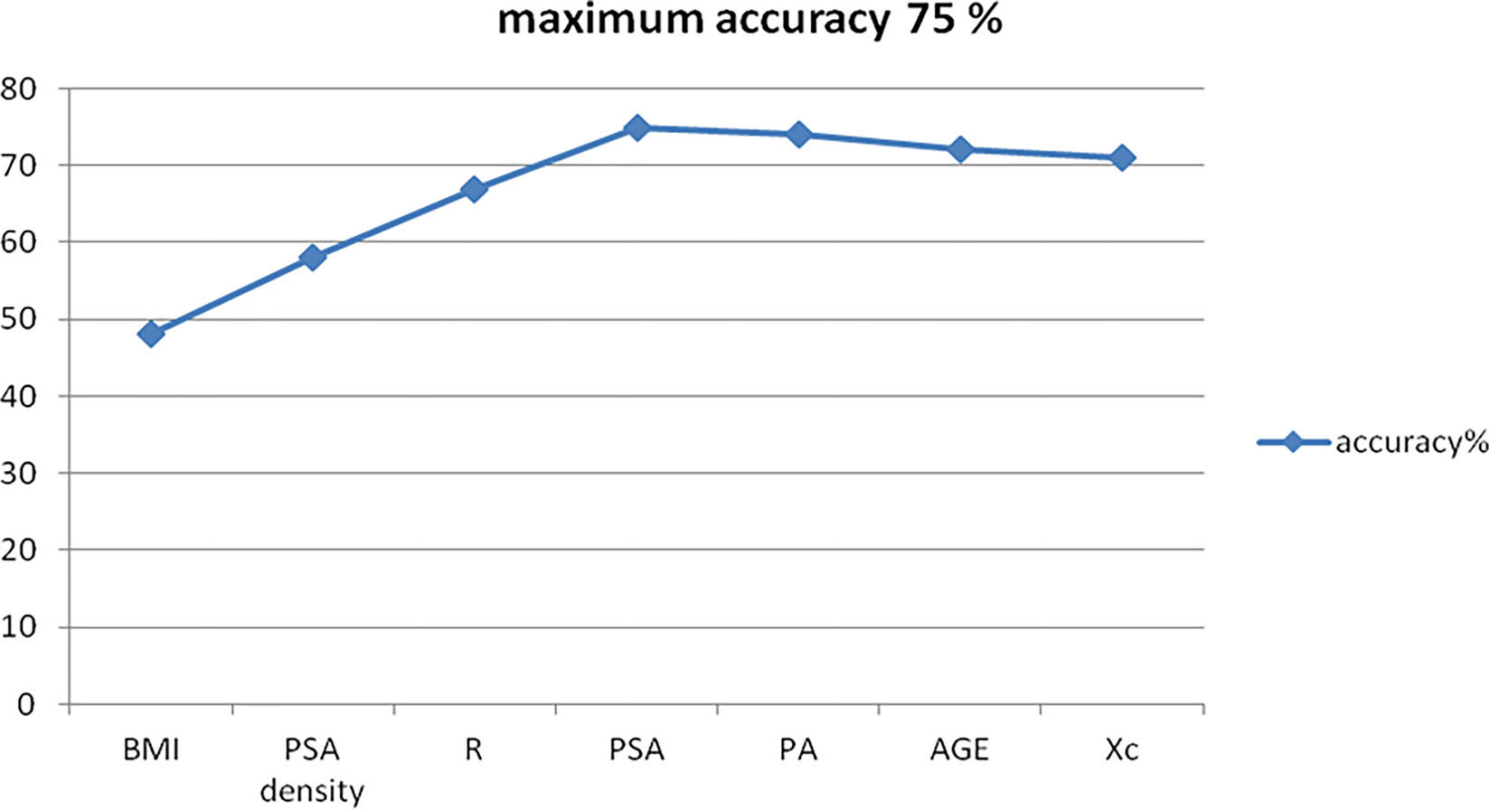
BIA test accuracy for the diagnosis of prostate cancer.

The average prediction accuracy achieved is shown in **Figure 2**, with a final score as of 75.00% (**Figure 4**).

This was obtained mathematically combining the following four parameters that were identified as clinically relevant: BMI, PSA density, R, and PSA. A comprehensive, ranked clinical feature list for this decision support system is reported in **Table 8**, while the corresponding confusion matrix is in **Table 9**. Sensitivity and specificity of the PCa prediction *vs.* BPH were 63.33% and 83.75%, respectively. The PPV and NPV were 74.51% and 75.28%, respectively. It is worthwhile noting that the resistance, averaged between the right and left prostate lobes, is one of the most informative features and gives a significant contribution to achieve the 75.00% of accuracy.

**Table 8:** Comprehensive ranked clinical feature list of the most accurate subset of features: BMI, PSA density, RES, and PSA.

BMI, PSA density, R, PSA	Cancer	BPH
**Cancer predicted**	**63.33%**	16.25%
**BPH predicted**	36.67%	**83.75%**

Bold values indicate significant p-values lower than 0.05.

**Table 9:** Confusion matrix of the most accurate sub-set of features excluding BIA parameters.

BMI, PSA density, PSA	Cancer	BPH
**Cancer predicted**	**51.67%**	28.75%
**BPH predicted**	48.33%	**71.25%**

BMI, Body Mass Index; PASD, prostate-specific antigen density; PSA, prostate-specific antigen.

Bold values indicate significant p-values lower than 0.05.

Importantly, as a counterproof, we obtained a significant decrease in the PCa prediction accuracy of 62.86% while repeating the same SVM-based computational procedure using a feature set that does not include the three biometric measures. As expected, the most informative subfeatures set included BMI, PSA density, and PSA.

## Discussion

BIA of different tissues was originally investigated by Geddes and Baker in the 1960s (22). They carried out an electrical measurement on living tissues demonstrating different values of resistivity. From that period, the BIA test have been used for various purposes such as the lean and fat body mass calculation and other medical applications like skin and breast cancer diagnosis (23–25). Halter et al. measured electrical properties of “*ex vivo*” prostate tissues with the aim of future applications for PCa noninvasive diagnosis. They realized that PCa, BPH, nonhyperplastic glandular tissue, and stromal tissue had different conductivity at all frequencies while mean cancer permittivity was significantly greater than that of benign tissues at high frequencies (15). Other authors demonstrated that best results for cancer diagnosis by BIA test were obtained by measuring the tissue phase angle. Low phase angle suggests cell death or decreased cell integrity, whereas higher phase angle suggests healthy cell (26, 27). A low phase angle has been associated with an impaired outcome in tumor diseases such as pancreatic cancer, colorectal cancer, and lung cancer (6, 7, 15). Tyagi et al. recently demonstrated that low phase angle values measured by BIA test allow for discriminating PCa patients from matched controls and those with advanced stage and high-risk PCa in particular. They investigated a group of subjects using the BIA electrode placement on the right upper and right lower limb. On the other hand, all PCa-diagnosed subjects had a total PSA increased values and other concomitant diseases excluded to avoid the risk of false-positive results (10). Similarly, Khan developed a new composite impedance metrics method with a nine-electrode microendoscopic probe. This novel device was tested on “*in vivo*” and “*ex vivo*” prostate tissue either intraoperatively or after the prostate removal in patients who underwent surgery for PCa or BPH. The results obtained demonstrated a predictive accuracy of 90.79% for PCa (11). For these reasons, we provided an alternative electrode placement and a restricted locoregional electric field in order to improve the BIA test sensitivity and specificity and reduce possible confounding factors. The finger probe allows the obtainment of the tissue resistance, reactance, and phase angle measurements directly from the prostate gland surface through a restricted electric field generated into the pelvic bone girdle. However, prostate tissue presents an extreme variability of electrical absorption due to its water and/or stromal content and the presence of microcalcifications in its tissue context with subsequent false positive results.

Our results demonstrated that the finger probe is a promising, reliable, and easy-to-use tool to improve the accuracy of PCa noninvasive diagnosis together with other standard clinical parameters. In patients where PCa was diagnosed in both prostate lobes (i.e., 65% of cases), BIA PA were found significantly different between the right and left side, while seemed to be comparable when PCa was diagnosed in a single lobe. Our experimental evidences on BIA phase angles do not replicate previous findings reported in (9). This may be justified by the presence of more represented stromal tissue and/or calcifications inside the gland, as well as by the normalization procedure that we have performed prior to the statistical analyses. All BIA measures including R, Xc, and PA, in fact, were normalized by dividing their value by the prostate volume estimated during the TRUS examination to avoid biases. Without normalization, patients with BPH and with PCa vs. healthy controls showed significant differences in terms of BIA PA (*p* = 0.006 and 0.003, respectively), therefore confirming previous observations by Tyagi et al. (10). BIA resistance values were lower in patients with PCa although, taken alone, it seemed to be unable to differentiate cancer from noncancer patients, while it was significantly different between healthy subjects and the BPH group. BIA reactance values were significantly different between healthy subjects and patients, although taken alone were not significantly different between BPH and PCa patients.

In this sense, likewise for the PSA alone, the BIA test failed to differentiate subjects with clinical suspicion of PCa and prospectively missed the intent of avoiding unnecessary biopsies. Nevertheless, when combined with the other standard clinical parameters including patients**’** PSA and PSA density, BIA test provided meaningful information for discerning between PCa and BPH patients with an accuracy as high as 75% at a single patient level.

Our results indicate a good PCa prediction using a combination of the following clinical features: BMI, Age, PSA, and PSA density. In this case, sensitivity and specificity are lower than the ones associated with a combination of BMI, PSA density, R, and PSA, thus demonstrating the significant clinical information associated with BIA test.

Study limitations include the limited amount of data, especially gathered from healthy volunteers, the nonage-matched group taken as negative control due to the increased risk of developing prostate diseases in the advanced age and a fixed range of 50 mHz frequency band for the BIA.

The proposed BIA test is a cheap, easy-to-perform method helpful for the multifeature clinical and noninvasive detection of prostate cancer and may be also able to decrease the number of unnecessary biopsies. The use of a novel transrectal “finger-probe” allows to do the BIA test with a minimal discomfort for the patient, contributing to an accuracy as high as 75% for the PCa vs. BPH prediction when properly combined with BMI, total PSA, and PSA density. Interestingly, the test can be easily repeatable. Further studies by varying the BIA tester voltage frequency are necessary to improve the BIA test efficacy. The cheaper cost of the method in comparison with mMRI may be immediately attractive for low-income countries.

## Author’s Note

Preliminary version of the manuscript has been previously preprinted at www.BiorXiV.org (https://doi.org/10.1101/2020.02.12.943829), and partial results are previously presented as an abstract at the European Urological Association Congress in Barcelona (European Urology Open Science March 2019, volume 18, issue 1, page e1810).

## Data Availability Statement

The raw data supporting the conclusions of this article will be made available by the authors, without undue reservation.

## Ethics Statement

The studies involving human participants were reviewed and approved by Internal Review Committee (IRC) (approval 1251/2017). The patients/participants provided their written informed consent to participate in this study.

## Author Contributions

Conceptualization: RB. Data curation: TV. Formal analysis: AG, ES, and GV. Investigation: JD and TV. Methodology: RB. Project administration: RB. Resources: RB, VF, and ES. Software: GV, AG, and ES. Supervision: RB, GV, and VF. Validation: RB and GV. Visualization: TV and AG. Writing original draft: RB and GV. Writing review edition: VF. All authors contributed to the article and approved the submitted version.

## Conflict of Interest

The authors declare that the research was conducted in the absence of any commercial or financial relationships that could be construed as a potential conflict of interest.

## Publisher’s Note

All claims expressed in this article are solely those of the authors and do not necessarily represent those of their affiliated organizations, or those of the publisher, the editors and the reviewers. Any product that may be evaluated in this article, or claim that may be made by its manufacturer, is not guaranteed or endorsed by the publisher.
